# Elevated Peripheral Blood Plasma Concentrations of Tie-2 and Angiopoietin 2 in Patients with Neuroendocrine Tumors

**DOI:** 10.3390/ijms13021444

**Published:** 2012-01-31

**Authors:** Gabriela Melen-Mucha, Agata Niedziela, Slawomir Mucha, Ewelina Motylewska, Hanna Lawnicka, Jan Komorowski, Henryk Stepien

**Affiliations:** 1 Department of Immunoendocrinology, Chair of Endocrinology, Medical University of Lodz, Dr Sterling Str. No. 3, 91-425 Lodz, Poland; E-Mails: gabriela.melen-mucha@umed.lodz.pl (G.M-M.); agatazn80@wp.pl (A.N.); ewelina.motylewska@umed.lodz.pl (E.M.); hanna.lawnicka@umed.lodz.pl (H.L.); 2 Department of Clinical Endocrinology, Chair of Endocrinology, Medical University of Lodz, Dr Sterling Str. No. 3, 91-425 Lodz, Poland; E-Mails: mucha.sla@wp.pl (S.M.); jan.komorowski@umed.lodz.pl (J.K.)

**Keywords:** angiogenic factors, NET patients, Ang-1, Ang-2, Tie-2

## Abstract

**Background:**

Gastro-entero-pancreatic/neuroendocrine (NET) tumors are highly vascularized neoplasms. However, our knowledge concerning circulating levels of the angiogenic factors in NET patients still remains insufficient.

**Methods:**

The aim of this study was to measure plasma concentrations of VEGF, angiopoietin 1 (Ang-1), angiopoietin 2 (Ang-2), soluble Tie-2, endostatin, osteopontin (OPN) and chromogranin A (CgA) in 36 NET patients and 16 controls.

**Results:**

Only the plasma concentrations of Tie-2 and CgA were higher in NET patients as compared to controls. These levels were within the reference range in controls; however one control demonstrated slightly elevated Tie-2 and 4 elevated CgA. Similarly, in the subgroup of patients with carcinoid syndrome, only Tie-2 and CgA concentrations were higher than those in patients with non-functioning NETs. In turn, in the subgroup of metastatic patients, only Ang-2 levels were higher than in those with localized disease. A positive correlation was found between Ang-2 and Tie-2 levels in metastatic patients and between Ang-1 and Tie-2 in localized NETs.

**Conclusions:**

The plasma concentration of Tie-2 is proposed as an additional marker for NET patients and seems to be similarly effective as the currently used CgA level. Moreover, higher plasma levels of Ang-2 together with the positive correlation between Ang-2 and Tie-2 levels in metastatic subjects, implies that cases with a Tie-2 level above the upper limits, together with higher level of Ang-2 seem to be highly predictive of metastases.

## 1. Introduction

Tumor angiogenesis is a highly complicated process consisting of several steps, known as the angiogenesis cascade, regulated by many pro- and antiangiogenic factors, both of endogenous and exogenous origin [1,2]. The group of endogenous angiogenic factors comprise, among others, the family of vascular endothelial growth factors (VEGFs) and their receptors (VEGF-A, VEGF-B, VEGF-C, VEGF-D and VGFR-2, VEGFR-3), the family of angiopoietins (Ang) and their receptors (Ang-1, Ang-2, Ang3/4 and Tie-1, Tie-2), other growth factor families, as well as endostatin and osteopontin (OPN). Some of these angiogenic factors are responsible for early activation of the angiogenic cascade, in which the VEGF signaling pathway acts as the most important early regulator of angiogenesis. The angiopoietin signaling pathway controls the later stages of the angiogenic cascade related to vessel maturation and quiescence.

Gastro-entero-pancreatic/neuroendocrine (NET) tumors are known to be highly vascularized neoplasms. However, our knowledge concerning circulating levels of the angiogenic factors in NET patients is rather weak and inconsistent. Since the incidence rate of these tumors has grown rapidly in the last two decades, this information seems to be very important not only for diagnostic purposes, but also for changes in the therapeutic options. The high vascularization of NETs suggests that the stromal cells play a role in tumor development.

Angiopoietin 1 (Ang-1) is a member of the angiopoietin family of growth factors. It plays a crucial role in mediating the reciprocal interactions between the endothelium, surrounding matrix and mesenchyme. It mediates blood vessel maturation and stability. Ang-1 binds and activates the Tie-2 receptor tyrosine kinase, which is expressed on the endothelial and lymphatic endothelial cells and participates in the regulation of angiogenesis and lymphangiogenesis procesess. Angiopoietin 2 (Ang-2) competes with this binding, thus blocking receptor phosphorylation and counteracting the blood vessel maturation and stability mediated by Ang-1 [3]. Its function, however, may be context-dependent. In the absence of angiogenic inducers such as VEGF, Ang-2-mediated loosening of cell-matrix contacts may induce endothelial cell apoptosis with consequent vascular regression. In concert with VEGF, Ang-2 may facilitate endothelial cell migration and proliferation, thus serving as a proangiogenic signal. It is known that Ang-2 regulates tumor angiogenesis in cooperation with VEGF, as well as with Ang-1, through Tie-2-dependent and independent pathways [4].

Endostatin, a 20 kDa fragment of type XVIII collagen inhibits angiogenesis and the growth of many tumors [5]. It has been demonstrated that endostatin prevents the activation of the angiogenic process and can also induce the regression of some established tumors [6]. In contrast to VEGF, circulating levels of endostatin correlate inversely with cancer angiogenesis and relapse-free survival time [7]. Although some studies indicate that the antitumor effect of endostatin can be diminished by VEGF [8], contradictory results showing either a positive correlation between these factors [9] or no correlation [10] have also been published.

Osteopontin belongs to the small integrin-binding ligand N-linked glycoprotein SIBLING family [11]. Its putative functions include roles in bone metabolism, immune response regulation, wound healing and tumor progression [12]. Growing evidence supports its role as a potential prognostic factor for various human cancers. OPN is expressed on human carcinomas, such as breast cancer, where it may have adhesion/migration functions in promoting invasion and metastasis [13]. Moreover, elevated OPN levels have been detected in cancer patients, including those with malignant mesothelioma [14].

A few studies have attempted to assess the blood concentrations of angiogenic factors in NET patients, but none provide information concerning such a wide panel of factors as that examined in our study. The data from a German study collected from 90 NET patients revealed increased Ang-2 serum concentration in examined NET subjects as compared to healthy controls. Higher Ang-2 levels were observed in metastatic as compared to local stages, which implies that Ang-2 can be applied as a prognostic marker [15]. Spanish data obtained from 42 NET patients showed enhanced serum concentrations of Ang-1, Ang-2 and soluble Tie-2 as compared to a 27-person control group, and revealed that Ang-2 and Tie-2 levels were higher in patients with metastases compared to those without [16]. In turn, data from a UK study on 47 NET patients revealed that serum Ang-2 was elevated in NET subjects, but not Ang-1. The Ang-2 level was higher in patients with metastases as compared to those with local disease [17]. Russian data revealed lower endostatin levels in benign NETs than in malignant cases [18]. The aim of the study was to search for new auxiliary diagnostic markers for patients with neuroendocrine tumors based on the analysis of six circulating angiogenic factors in plasma.

## 2. Results and Discussion

### 2.1. Patients

The characteristics of 36 patients with NETs including data concerning age, sex, metastases, hormonal function, localization of primary tumor and somatostatin analog therapy is presented in detail in Table 1.

#### 2.1.1. Angiogenic Factors Levels in Patients with NETs and Healthy Controls

**Figure 1:**
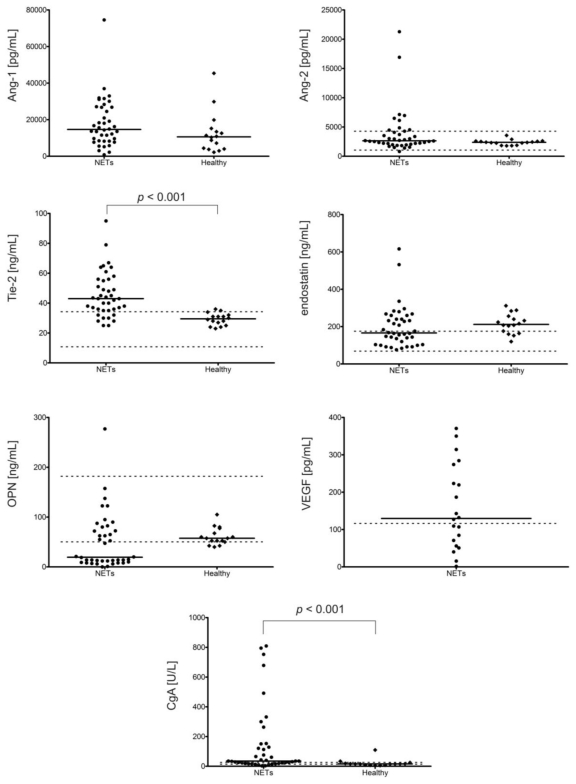
Plasma angiogenic factors (Ang-1, Ang-2, Tie-2, endostatin, osteopontin (OPN), VEGF) and CgA levels in NETs patients and controls (16 volunteers without VEGF measurements) assessed with ELISA. Solid horizontal lines correspond to the medians of plasma levels; in turn, two dashed lines (upper and lower) represent the limits of reference values for these factors in EDTA plasma. The reference range for plasma Ang-1 levels is not given by kit producer (R&D Systems). Only the plasma levels of Tie-2 (*p* < 0.001) and CgA (*p* < 0.001) were higher in NET patients as compared to controls (Mann-Whitney *U* test).

Patients with NETs had significantly higher plasma levels of Tie-2 (*p* < 0.001) and CgA (*p* < 0.001) as compared to healthy controls (Figure 1).

The median of the Tie-2 levels was more than 40% higher than that of the controls while CgA was more than 2 times higher. The levels of these factors in the 16 subjects within the control group were within reference range, except for 1 subject with slightly elevated Tie-2, and 4 subjects with elevated CgA levels. In turn, the levels of Tie-2 and CgA remained within the reference range in 10 and 9 NET patients respectively (only one patient had these two factors within normal range). Hence, Tie-2 level would seem to be an additional diagnostic marker for NET patients and seem to have a similar effectiveness as the currently-used CgA levels. In addition, in NET patients, a few positive correlations were found between Tie-2 and Ang-2 (*p* < 0.01), Ang-2 and CgA (*p* < 0.05), as well as endostatin and OPN (*p* < 0.001). Figure 1 shows scatter plots of the angiogenic factor levels with their boundaries of reference range and medians in the NET group and healthy controls.

Moreover, the medians of Ang-1 and Ang-2 levels were also higher in the NET group; in turn, the medians of endostatin and OPN were lower than that of the controls, however, the differences did not reach the level of statistical significance.

The main reason why the markedly different median values were not statistically significant is the great diversity of the obtained results, especially in the NET group. For example, the range of values in the NET group for Ang-1 levels was 836 pg/mL (min) and 74573 pg/mL (max) *vs.* 2192 pg/mL (min) and 45396 pg/mL (max) in controls; and the range for CgA levels in the NET group was 2.4 U/L (min) and 809.4 U/L (max), and 4.4 U/L (min) and 109.6 U/L (max) in controls.

In controls, a negative correlation was observed between age and CgA (*p* < 0.05), and between age and Tie-2 levels (*p* < 0.01), but a positive correlation between age and endostatin concentrations (*p* < 0.05). In controls, the level of CgA positively and strongly correlated with OPN (*p* < 0.001) and weakly with Tie-2 (*p* < 0.05). Additionally, of all the examined factors in our control group, only the levels of Ang-2 and Tie-2 (except for one case) did not go beyond the boundaries of reference standards given in R&D Systems materials. OPN levels were below the lower limits in 3 out of 16 healthy volunteers, whereas endostatin levels exceeded the upper limits in 13 out of 16 healthy controls, which suggest the need to reassess and re-establish new standards for plasma concentrations of endostatin (Figure 1).

#### 2.1.2. Angiogenic Factors Levels in Patients with Carcinoid Syndrome in Comparison to Non-Functioning NETs

**Figure 2:**
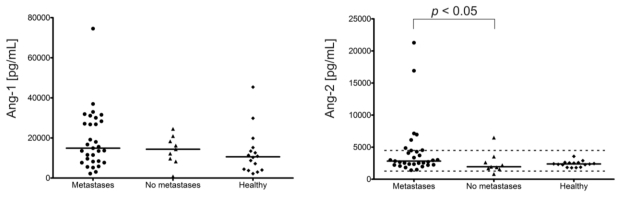

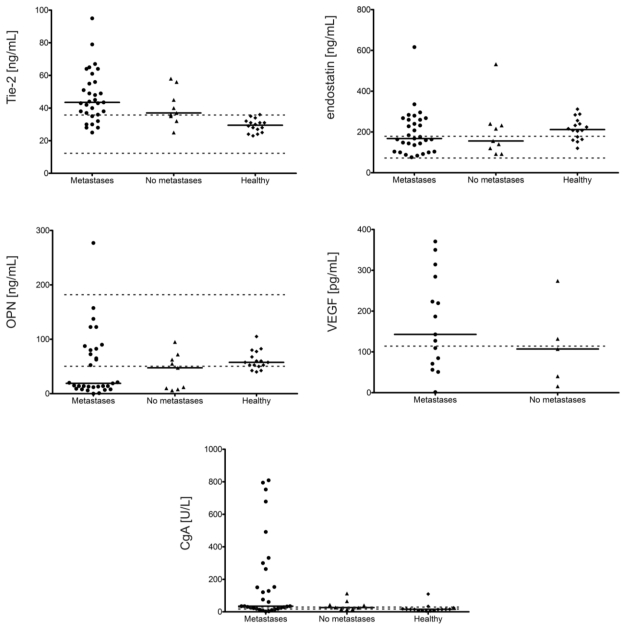
Plasma angiogenic factors (Ang-1, Ang-2, Tie-2, endostatin, OPN, VEGF) and CgA levels in patients with carcinoid syndrome (7 pts), non-functioning NETs (29 pts) and controls (16 volunteers without VEGF measurments) assessed with ELISA. Solid horizontal lines correspond to the medians of plasma levels; in turn, two dashed lines (upper and lower) represent the limits of reference values for these factors in EDTA plasma. The reference range for plasma Ang-1 levels is not given by kit producer (R&D Systems). Only the plasma levels of Tie-2 (*p* < 0.05) and CgA (*p* < 0.001) were higher in the subgroup of patients with carcinoid syndrome than those in patients with non-functioning NETs (*post hoc* Kruskal-Wallis test).

NET patients with carcinoid syndrome (7 pts) had significantly higher levels of Tie-2 (more than 40%, *p* < 0.05) and CgA (more than 20 times, *p* < 0.001) as compared to patients with non-functioning tumors (29 pts) (Figure 2). Among the patients with carcinoid syndrome none had Tie-2 or CgA levels within the reference range. The median levels of factors other than endostatin, OPN and Ang-1 were also higher; the median Ang-2 level, for example, was almost 2 times higher, but the differences were insignificant. Moreover, a positive correlation was found between endostatin and OPN (*p* < 0.05 in NETs with carcinoid syndrome, *p* < 0.01 in non-functioning NETs) in both subgroups, and also between Ang-2 and Tie-2 in non-functioning tumors (*p* < 0.05).

#### 2.1.3. Angiogenic Factors Levels and Stage of Disease

**Figure 3:**
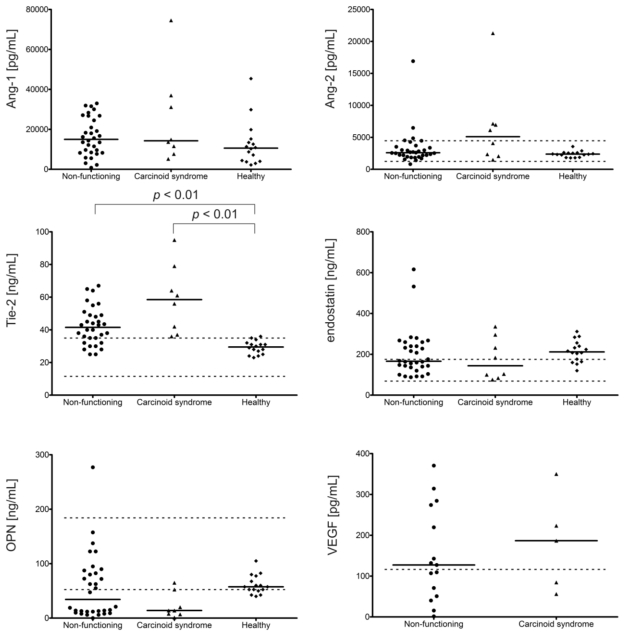

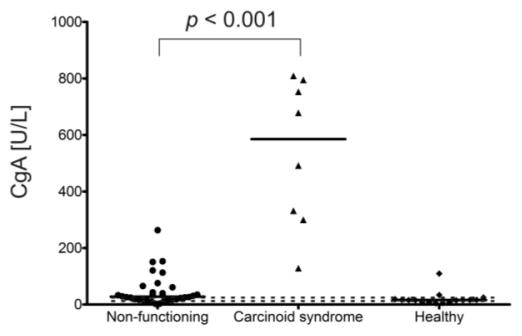
Plasma angiogenic factors (Ang-1, Ang-2, Tie-2, endostatin, OPN, VEGF) and CgA levels in metastatic NETs patients (27 pts), NET patients with localized disease (9 pts) and controls (16 volunteers without VEGF measurments) assessed with ELISA. Solid horizontal lines correspond to the medians of plasma levels; in turn, two dashed lines (upper and lower) represent the limits of reference values for these factors in EDTA plasma. The reference range for plasma Ang-1 levels is not given by kit producer (R&D Systems). In the subgroup of metastatic patients, only Ang-2 (*p* < 0.05) levels were higher than in those with localized disease (*post hoc* Kruskal-Wallis test).

In patients with metastatic disease (27 pts), only Ang-2 levels were significantly higher than in NET patients with localized disease (9 pts) (*p* < 0.05): the median value being more than 45% higher (Figure 3). However, there was no correlation between the presence of metastases and Ang-2 levels. In metastatic NETs, a positive correlation was found between Ang-2 and Tie-2 levels (*p* < 0.05), whereas in localized NETs a positive correlation was observed between Ang-1 and Tie-2 (*p* < 0.01). In both subgroups, a positive correlation was seen between endostatin and OPN (*p* < 0.01). The median levels of other factors, except for OPN, were also higher in advanced disease but the differences were statistically insignificant.

#### 2.1.4. Angiogenic Factor Level and NET Types

There were no statistically significant differences between medians of the examined levels of factors in patients with foregut, midgut and hindgut NETs, however some correlations were observed in each subgroup. In foregut patients, a negative correlation was seen between age and OPN (*p* < 0.05) and between Ang-1 and endostatin (*p* < 0.05) while a positive correlation was seen between CgA and Ang-1 (*p* < 0.01), endostatin and OPN (*p* < 0.01) and Ang-2 and Tie-2 (*p* < 0.05); in midgut patients, only a positive correlation was observed between Ang-1 level and VEGF to endostatin ratio (*p* < 0.001); finally, in hindgut patients, a negative correlation was found between Ang-1 and Tie-2 (*p* < 0.05) and three positive correlations were found (*p* < 0.05) between endostatin and Ang-2 to Tie-2 ratio, OPN and Ang-2 to Tie-2 ratio, and between Tie-2 level and Ang-2 to Ang-1 ratio.

Ordering the median value of these factors from largest to smallest, a certain regularity can be observed. For Tie-2, the highest levels were in the midgut, then the foregut and then the hindgut (53 *vs.* 40 *vs.* 38 ng/mL respectively). For Ang-2, the highest levels were noticed in the midgut, lower in the hindgut and the lowest in the foregut (3555 *vs.* 2823 *vs.* 2352 pg/mL respectively). The medians for Ang-1 in foregut and midgut NETs (14,373 and 14,651 pg/mL respectively) were very close and higher than in the hindgut (12,191 pg/mL). The highest median of VEGF levels were observed in foregut NETs, lower in the midgut and the lowest in the hindgut (163 *vs.* 132 *vs.* 61 pg/mL respectively). For endostatin and OPN, the highest to the lowest medians occurred in the opposite order to that of VEGF; both factors had the highest median in the hindgut and the lowest in the foregut (endostatin: 240 *vs.* 196 *vs.* 168 ng/mL; OPN 63 *vs.* 36 *vs.* 19 ng/mL respectively).

#### 2.1.5. Changes in Angiogenic Factors Levels in Selected NET Patients, Who Had Two Blood Samples Taken

Double blood sampling in 4 out of 36 patients allows the changes in concentrations of examined angiogenic factors to be analyzed with passing time and, in 3 patients, after the introduction of somatostatin analog therapy. The characteristics of these 4 patients, including data concerning sex, age, metastases, hormonal function, the type of NET, time of the introduction of somatostatin analog therapy, their dosages and time of treatment, together with the levels of examined angiogenic factors and the changes in their levels expressed in percent, is presented in detail in Table 2.

In one of these patients (69-year-old woman, Table 2), a dramatic decrease in the levels of all examined factors except OPN was observed 1 month after the first injection of octreotide LAR, at almost unchanged clinical status. In the next one (57-year-old man, Table 2) with significant improvement in clinical status observed after 7 months of lanreotide autogel therapy (despite of an increase of CgA levels), the levels of Ang-1, OPN and endostatin decreased, whereas of Ang-2 and Tie-2 increased. In another one (47-year-old man, Table 2), the second sampling was performed just before his death, at very poor clinical status after 1 year and 1 month of octreotide therapy and revealed a drop in Ang-2, Tie-2, OPN and VEGF levels together with a rise in Ang-1 and endostatin. In the last patient (57-year-old man, Table 2), the blood was taken for the second time after almost 2 years since the removal of gastric GIST, at the time when partial regression of NET foci together with disappearance of symptoms were noticed, and revealed a huge rise in the levels of all the factors except Tie-2.

#### 2.1.6. Discussion

This is the first study assessing the levels of so many angiogenic factors (Ang-1, -2, Tie-2, endostatin, OPN and VEGF) in patients with NETs, in order to ascertain the profile of these factors and their mutual correlation with the hope of finding among them new additional diagnostic markers for these unpredictable tumors. In some ways our data is unique because the levels of these factors have been evaluated in plasma instead of serum to avoid the influence of platelets.

The levels of these factors differ significantly between serum and plasma. The reference ranges for these factors are much narrower, and the upper limits of the normal values much smaller, in plasma than in serum (the plasma values for Ang-2, Tie-2 are half the serum values and the VEGF upper limit is almost 6 times smaller in plasma than serum) apart from those of OPN, whose serum values achieve about 50% of the plasma values (artificially decreased by thrombin proteolytic cleavage). This regularity is caused by platelets, which have the ability of selective uptake of all angiogenic factors in cancer-bearing individuals [19]. As a result, these factors are released from platelets into the serum during platelet activation when the clot is forming. Thus, the plasma levels of these factors reflect circulating protein content, while serum levels reflect the sum of plasma content and the amount of factors released from platelets during their activation.

However, the released amount of angiogenic factors from platelets depends on a number of variables. Firstly, the number of platelets is important, and hence, some authors who are aware of this phenomenon, present serum levels of these factors with a correction for platelet count based on Adams *et al.* [20]. Secondly, the time of platelet activation can have an influence. For example, during the clot formation in a clot-activator tube, the amount of the released VEGF increases with time and reaches a plateau after 60 min. Hence, in some papers, the blood samples were allowed to clot for 2 h [21]. Finally, the type of platelet activator, such as ADP, thrombin or clot-activator tube, and the type of angiogenic factors, for example VEGF or bFGF, can have an influence [19]. Moreover, the released amount of angiogenic factors from platelets can be also changed by antiplatelet drugs such as aspirin [22] and perhaps by other factors.

The influence of even a small number of platelets in plasma prepared according to standard procedures (recommended by the National Committee for Clinical Laboratory Standards) is highlighted by the instructions given in R&D Systems materials, where the company advises an additional centrifugation step for complete platelet removal (platelet-free plasma) to avoid variable and irreproducible results. Moreover, the company does not give the reference range for Ang-1 concentrations in plasma prepared according to the typical procedure, hence the lack of two horizontal lines representing the boundaries of the reference range on our figures.

Therefore, as the platelets seem to be the richest source of the aforementioned substances and can selectively take up or release them in an unpredictable way, the plasma levels of the examined factors may be superior and, undoubtedly, more stable than their serum concentrations.

In our study, of all the factors examined, only the plasma levels of soluble Tie-2 and CgA were elevated in patients with NETs as compared to the healthy controls. Therefore, we would suggest Tie-2 peripheral blood plasma concentration as new additional diagnostic marker for NET patients. In our opinion, it seems to be similarly effective as the currently used CgA level. The number of false positive and false negative results detected in our study favors even Tie-2 over CgA levels; only one person from healthy controls had an elevated (above upper limit of normal) level of Tie-2, as opposed to 4 with an increased CgA level. Additionally, while Tie-2 levels were within reference range for 10 NET patients, CgA levels remained within the reference range for 9 NET patients.

In other cited studies concerning NET patients, the levels of these factors were evaluated exclusively in serum, which makes the comparison rather difficult. Moreover, it has been shown that the levels of VEGF [21] or endostatin [22] do not correlate in serum and plasma.

Despite this inconsistency a survey of the literature reveals that our results are concordant with data from Spain (the only study in which Tie-2 levels were measured in NET patients) in which elevated levels of Tie-2 were observed in NET patients when compared to the controls [16]. The authors also noticed elevated levels of Ang-1 and Ang-2, which were not shown in our study. In the Spanish report, Tie-2 levels in the healthy subjects were found to overlap those of NET patients much more frequently, although there is no precise description of the overlapping data in the cited paper [16]. Although the levels of Tie-2 in both this and the Spanish studies were assessed by the same experimental kit (R&D Systems), the results did not reveal the expected differences between serum and plasma levels (higher levels in serum than in plasma); in the Spanish study, the serum Tie-2 level in NET patients was 25.9 ± 9.5 (X ± SD) ng/mL *vs.* controls 17.1 ± 4.1 ng/mL whereas in our study, the plasma Tie-2 level in NETs was 43 (25–95) ng/mL (median and range) *vs.* control 30 (23–35) ng/mL.

In contrast to our results, the Spanish report also revealed higher Tie-2 and Ang-2 levels in patients with metastases as compared to those without. In our patients’ samples, only the plasma Ang-2 levels were higher in metastatic as compared to local NET subjects. This finding is consistent with the English [17] and German [15] studies, where Ang-2 levels were assessed in serum unlike to our study.

However, in all these studies, the results from both NET subgroups (metastatic and localized) overlapped. In this event, the positive correlation between Ang-2 and Tie-2 levels observed by us only for metastatic patients may be helpful in diagnostic evaluation. Cases with a combination of a Tie-2 level above the upper limits, together with a rather high level of Ang-2 seem to be highly indicative of metastases. In our study, 20 out of 27 patients with metastases had elevated Tie-2 levels. In turn, the concentrations of Tie-2 and CgA were within reference range only in 7 and 6 out of 27 metastatic patients respectively suggest an important role of Tie-2 in NET progression. Moreover, two positive correlations seen in our patients, one between Ang-2 and Tie-2 in metastatic patients, and the second between Ang-1 and Tie-2 in localized NETs, seem to be obvious, when taking into consideration the physiological role of these angiomodulators in angiogenesis.

Similarly, no expected differences regarding Ang-2 levels were observed between ours (plasma) and the other studies (serum), especially in control subjects, although the same kit from R&D Systems was used by all the authors. In our study, the median of Ang-2 levels in controls was 2380 pg/mL; in the study from England—2495 pg/mL; from Germany—2650 pg/mL and from Spain—1.7 ng/mL (arithmetic mean).

In our opinion, the differences between biological materials (plasma and serum) cannot be solely responsible for the presented discrepancies between this study and the others. Instead, it may well be due to the unusual heterogeneity of NET tumors. The recommendation from the consensus report of the National Cancer Institute that ”carcinoid tumors and pancreatic NETs should be examined separately” is still not taken into consideration in ours and other studies [23]. However, with this in mind, our study concerns mainly non-pancreatic NETs with only 2 pancreatic cases (6%), whereas pancreatic NETs were strongly represented in other reports: in the study from England, 17 out of 47 (36%); from Germany, 25 out of 42 (60%) and from Spain, 23 out of 42 patients (55%).

Moreover, some discrepancies between the Spanish and English data, for example, concerning Ang-1 levels, which are elevated in NET patients from Spain but not from England, can be caused by the difference in the number of platelets; although both studies measured the levels of this factor in serum, none gave any information about the platelet count.

Other data from our study did not reach the threshold of statistical significance, however the paucity of published data concerning the levels of angiogenic factors in NET patients demand that some interesting, in our opinion, observations be highlighted. Namely, the levels of angiogenic factors are not stable in NET patients and can be changed drastically even within a very short period of time (one month), as was observed in one of our patients. Most likely, these major changes, and the small decrease in VEGF concentration observed in our other patient, may be caused by somatostatin analog treatment, which is in accordance with on *in vitro* study showing the inhibition of VEGF secretion from neuroendocrine tumor cells by octreotide [24,25]. In addition, the endostatin and OPN levels in our studies were found to be rather low in NET patients in comparison to healthy controls, contradicting the findings of the other studies, which show elevated OPN levels in patients with mesothelioma [14] or endostatin in renal cell carcinoma patients [9] and others. Finally, our study is the first in the literature in which OPN levels were evaluated in patients with NETs.

## 3. Materials and Methods

### 3.1. Patients

Thirty-six NET patients were enrolled between May 2008 and February 2011 to the study. All the patients had neuroendocrine tumors as confirmed histologically by morphological assessment as well as by immunohistochemical analysis for chromogranin A (CgA), synaptophysin and, where it was possible, by Ki67 evaluation according to the 2000 WHO classification for gastrointestinal tract and from 2004 for the bronchopulmonary tract. All 36 cases were classified as well-differentiated neuroendocrine tumors or cancers. All these patients were diagnosed and treated at the outpatient endocrinology clinic and at the Department of Clinical Endocrinology, Medical University of Lodz, Poland. The study was approved by the by the Ethics Committee of the Medical University of Lodz. The written informed consent was obtained from all participants before blood sampling after a full explanation of the purpose of the study and the nature of all procedures.

Plasma samples from all patients were collected during routine examinations for CgA assessment; this was performed just before the drug injection in most patients treated with somatostatin analogs. In 4 out of 36 patients, plasma samples were collected twice at different time points due to important reasons which are supposed to influence upon the examined parameters, such as the introduction of somatostatin analog therapy. These patients are described in detail in the results section. The other data concerning these patients, such as age, sex, localization of the primary tumor, presence of metastases, hormonal function and somatostatin analog therapy were obtained from systemic review of the appropriate medical documentation. The control groups comprised 16 healthy volunteers (age- and gender-matched) enrolled in the same period. All of them are employees at the Chair of Endocrinology, Medical University of Lodz and had no previous history of cancer.

### 3.2. ELISAs

Venous blood samples were collected into vacutainer tubes supplemented with EDTA from all the patients and control volunteers. The samples were centrifuged at 1000xg for 15 minutes within 30 minutes of collection and stored at −20 °C. ELISA kits from R&D Systems (Quantikine Human VEGF, Quantikine Human Angiopoietin-1, Quantikine Human Angiopoietin-2, Quantikine Human TIE-2, Quantikine Human Endostatin, Quantikine Human Osteopontin) were used to measure the concentrations of the following angiogenic factors in the plasma samples: VEGF (assessed only at 20 NET patients due to financial limits), Ang-1, Ang-2, soluble Tie-2, endostatin and OPN. The assay Quantikine Human VEGF is designated to measure the levels of VEGF_165_ in various biological materials. It is a proangiogenic form of VEGF A in contrast to its anti-angiogenic splice variant—VEGF_165b_. All these assays employ the quantitative sandwich enzyme immunoassay technique by using a microplate precoated with a monoclonal antibody specific to an appropriate factor. Chromogranin A was measured by a Dako ELISA kit (Chromogranin A ELISA kit), which utilizes rabbit antibodies specific to a 23 kDa *C*-terminal fragment of human chromogranin A. It was decided to evaluate the plasma levels of these factors to avoid the well-known effect of platelets on the levels of these factors in serum [19]. In addition, it is recommended that osteopontin should be assayed in plasma in order to avoid cleavage by thrombin during the clotting process.

### 3.3. Statistical Analysis

Statistical analysis was performed using Statistica 9 Software [26]. Since the obtained data was not normally distributed (the Shapiro-Wilk test), the non-parametric Mann-Whitney *U* test (for two groups) or Kruskal-Wallis test (for several groups) were used to compare differences between examined groups. The Spearman correlation coefficient was used to analyze correlations between parameters. Differences were considered significant if *p* < 0.05. Moreover, a trial version of Graph Pad Software [27] was used to create all figures in order to present each separate result, which is not possible using Statistica 9 Software.

## 4. Conclusions

Summing up, enhanced peripheral blood plasma Tie-2 concentration seems to be an additional diagnostic marker for NET patients offering a similar effectiveness as the currently used CgA levels. Moreover, higher plasma levels of Ang-2 together with the positive correlation between Ang-2 and Tie-2 levels in metastatic subjects, implies that cases with a Tie-2 level above the upper limits, together with higher level of Ang-2 seem to be highly predictive of metastases.

## Figures and Tables

**Table 1:** The characteristics of patients enrolled to the study.

Patient Characteristics	
**Age**—median (range)	61 years (31–80)
Male—number of patients (median of age; range)	17 (57 years; 32–80)
Female—number of patients (median of age; range)	19 (66 years;31–76)
Patients with **metastatic disease**—number of patients (%)	27 (75%)
Patients **without metastases**—number of patients (%)	9 (25%)
Patients with **carcinoid syndrome**—number of patients (%)	7 (19%)
Patients with **non-functioning NETs**—number of patients (%)	29 (81%)
**Type of NETs**—number of patients (%)	
foregut	11 (31%)
midgut	12 (33%)
hindgut	6 (17%)
unknown or others	7 (19%)
**Localization of the primary tumor**—number of patients (%)	
lung	6 (17%)
thymus	1 (3%)
pancreas	2 (6%)
stomach	2 (6%)
small intestine and appendix	12 (33%)
rectum	6 (17%)
unknown or others	7 (19%)
**Concurrent SST analogs**—number of patients (%)	14 (39%)

**Table 2:** Changes in angiogenic factors levels in four NET patients, who had two blood samples taken.

Patient	Time of Two Blood Samplings & Changes	CgA (U/L)	Tie-2 ng/mL	Ang-2 (pg/mL)	Ang-1 (pg/mL)	End ng/mL	OPN ng/mL	VEGF (pg/mL)
♀ 69-year-old metastatic nonfunctioning midgut NET (small intestine)	**1**/before the first injection of 20 mg octreotide LAR (Novartis)	34.8	64	16921	13571	208	123	ND
	**2**/one month later, before the second injection	ND	43	3024	8228	160	123	ND
	**changes**		**↓30%**	**↓80%**	**↓40%**	**↓20%**	**0%**	

♂ 57-year-old metastatic midgut NET with carcinoid syndrome	**1**/before the first injection of 90 mg lanreotide autogel (Ipsen)	492	37	2062	13671	296	65	ND
	**2**/after 7 months of therapy (90 mg every 4 weeks) with well-controlled symptoms	678.5	42	4086	5231	232	52.5	ND
	**changes**	**↑40%**	**↑10%**	**↑100%**	**↓60%**	**↓20%**	**↓20%**	

♂ 47-year-old metastatic non-functioning NET of the left kidney	**1**/good clinical status, 2 months before the first dose of 20 mg octreotide LAR (Novartis)	75.7	38	3708	27102	92	14	370.6
	2/poor clinical status, just before death, after 1 year and 1 month of octreotide therapy (20 mg every 4 weeks) and after many resections of metastatic foci	60.8	25	3376	28362	100	13	314.3
	**changes**	**↓19%**	**↓34%**	**↓9%**	**↑5%**	**↑8%**	**↓**7%	**↓13%**

♂ 57-year-old metastatic nonfunctioning rectal NET and gastric GIST	1/just before GIST removal on octreotide therapy	11.1	48	1406	2217	144	6	ND
	2/after almost 2 years passed, without new GIST foci and with partial regression of NET metastatic foci, continuously treated with octreotide LAR for 3 years and 6 months (Novartis) 20 mg i.m. every 4 weeks	13.2	43.5	2823	7701	240	63	ND
	**changes**	**↑19%**	**↓10%**	**↑100%**	**↑350%**	**↑160%**	**↑1000%**	ND

CgA: chromogranin A; Tie-2: soluble form Tie-2; Ang-1, -2: angiopoetin 1, 2; End: endostatin, OPN: osteopontin, VEGF: vascular endothelial growth factor; ↓: decrease; ↑: increase; ND: not determined.
